# Papillary fibroelastoma of a mitral valve chordae, presenting with atypical chest pain and palpitation: A case report and the literature

**Published:** 2014

**Authors:** Shervin Ziabakhsh, Rozita Jalalian, Farzad Mokhtari-Esbuie

**Affiliations:** 1 Associate prof. of Cardiac Surgery, Cardiac Surgery Department, Fatemeh Zahra Hospital of Sari, Mazandaran University of Medical Sciences, Sari, Iran.; 2 Cardiovascular Department, Fatemeh Zahra Hospital of Sari, Mazandaran University of Medical Sciences, Sari, Iran.; 3 General Physician, Fatemeh Zahra Hospital of Sari, Mazandaran University of Medical Sciences, Sari, Iran.

**Keywords:** Papillary fibroelastoma, Cardiac tumor, Mitral valve chordae.

## Abstract

***Background: ***Primary intra cardiac tumors are rare. In this article, we present papillary fibroelastoma of mitral valve chordae.

***Case Presentation:*** A 35-year old man presented with atypical chest pain and palpitation. Physical examination and electrocardiogram were normal. Transesophageal echocardiography (TEE) revealed a mass of 1015 mm attached to chordae of anteromedial papillary muscle of mitral valve. The tumor was completely resected and the mitral valve chordae tendineae was preserved successfully. The pathological diagnosis was papillary fibroelastoma.

***Conclusion: ***In any patient with atypical chest pain and palpitation, valvular tumor should be considered in differential diagnosis.

Primary intra cardiac tumors are rare with a prevalence ranging from 0.0017 to 0.28% in various autopsy series and account for an only 0.3% of all open heart operations (1, 2). Papillary fibroelastomas (PFE) account for less than 10% of all cardiac tumors, representing the most common valvular and the second most common cardiac benign tumor following myxomas (3, 4). Papillary fibroelastoma is primary cardiac tumor. The size of this tumor varies from 2 mm to 70 mm (5, 6). Papillary fibroelastomas are seen as gelatinous masses with a characteristic "sea anemone" appearance due to the presence of multiple delicate papillary fronds. Immunohistochemical studies have introduced the concept of a virus-induced local growth, in which together with the microthrombus and valve degeneration theories further support that these are acquired rather than congenital lesions. Current explanations for mechanism of systemic embolization include direct embolism of pieces of the tumor itself versus mobilization of thrombi, as only a single layer of endothelium covers the highly thrombogenic matrix. There is some belief that cardiac fibroelastomas may even originate from prior thrombotic insults themselves (7-9). Most cases of PFE are asymptomatic and discovered incidentally during imaging before cardiac surgery (10, 11), but symptomatic presentation includes mostly myocardial ischemia, myocardial infarction and stroke (10-14). However, pulmonary embolism, congestive heart failure, near syncope, ventricular fibrillation and sudden death has also been reported (5, 15). In this report, we present a 35-year old man with papillary fibroelastoma of a mitral valve chordae, presenting with atypical chest pain and palpitation.

## Case presentation

**Figure 1:**
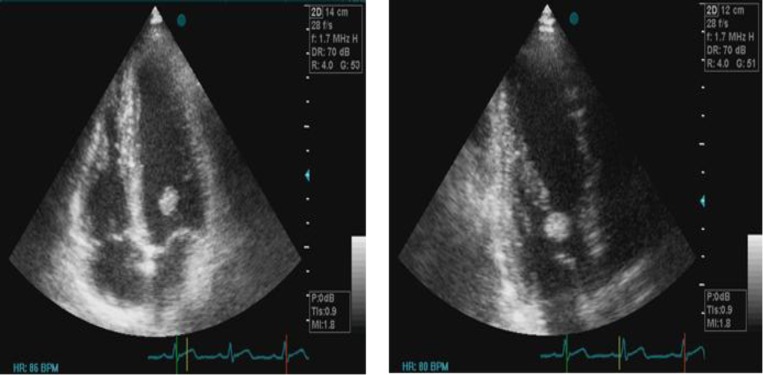
A, B Echocardiography (Four chamber and three chamber view) showed a bulbar mass of 10 15 mm attached at mitral valve anterior leaf let (a white arrow).

A 35-year old man was referred to our department for the evaluation of atypical chest pain and palpitation. The physical exam and electrocardiogram were normal and routine blood laboratory tests were unremarkable. Transthoracic echocardiography showed a mobile mass on mitral valve. However, the aortic valve and cardiac chambers were normal. There was no regurgitation of the mitral valve, and ejection fraction was normal. Transesophageal echocardiography (TEE) revealed a mass of 1015 mm attached to chordae of anteromedial papillary muscle of mitral valve (figure1). 

**Figure 2:**
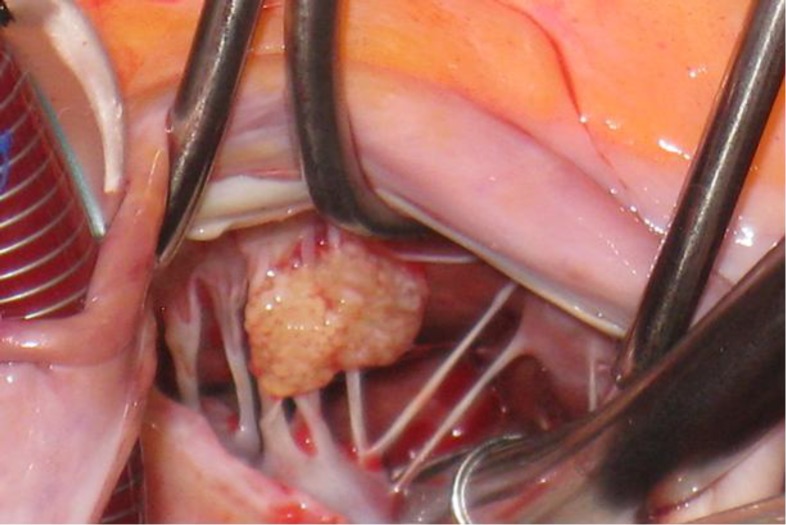
Macroscopic finding of papillary fibroelastoma with attachment to chordae tendineae of mitral valve. The tumor was lobulated, soft and yellowish in color

Blood culture, sedimentation rate, and testing for thrombophilia disorders were not remarkable. Based on its place and appearance, the working diagnosis of a cardiac papillary fibroelastoma was made. The patient underwent surgical resection of this mass. The heart was exposed through a median sternotomy. After appropriate cannulation, cardiopulmonary bypass was started, and then the mitral valve was reached through a trans-septal approach. The multilobular yellowish mass was seen attached to chordae of the anteromedial pappilary muscle of the mitral valve (figure 2). Tumor was completely resected from mitral valve chordae; fortunately, chordae tendinaes were preserved successfully without destruction. 

**Figure 3:**
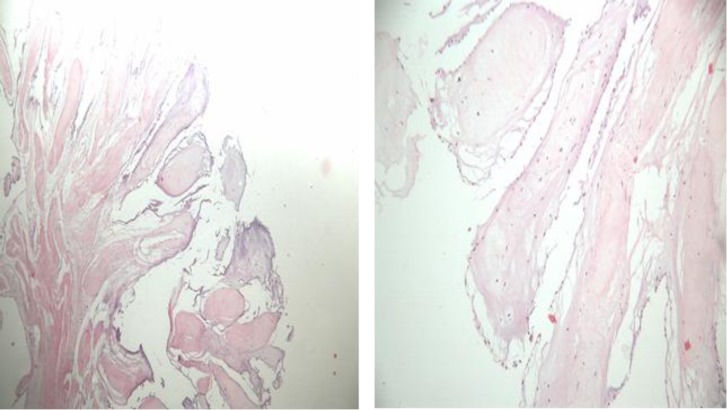
A, The Histological specimen in the hematoxylin and Eosin stain shows a benign lesion with multiple papillary fronds. B, The papillary fronds consist of hyalinized hypocellular stroma lined by flat endocardial cells

**Figure 4:**
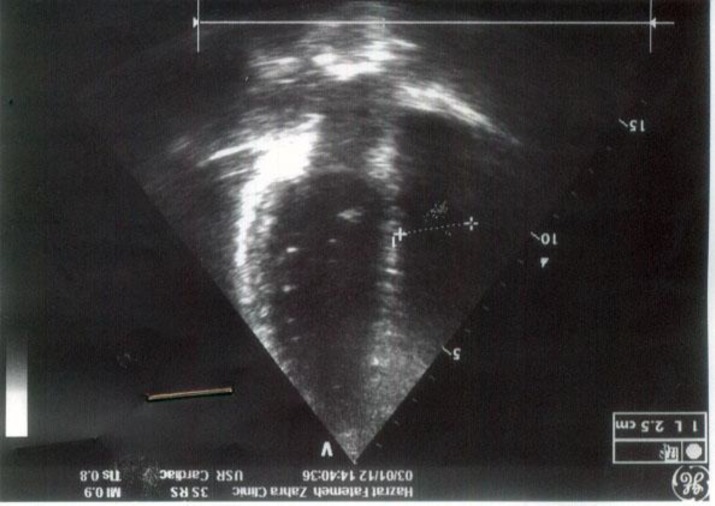
Echocardiography: Normal mitral valve without regurgitation, and no occurrence of tumor

The tumor was attached to three chordae tendinaes and was not pedanculated. The patient had a good weaning from the cardiopulmonary bypass and recovered well in the post-operative period. The postoperative TEE showed absence of residual tumor and normal mitral valvular function and there were no findings of mitral valvular regurgitation. The histological findings of the tumor were compatible with papillary fibroelastomas (figure 3). After 7 months of follow-up, the patient had no symptoms and repeated TTE showed normal mitral valve without regurgitation or recurrence of this tumor (figure4).

## Discussion

Primary intra-cardiac tumors are rare with a prevalence ranging from 0.0017 to 0.28% in various autopsy series (1, 2). Papillary fibroelastomas (PFE) account for less than 10% of all cardiac tumors. Although they are benign tumors; they can result in life-threatening complications such as myocardial infarction, cardiac arrest and stroke (12-14). Therefore, for early diagnosis of papillary fibroelastoma and prevention of severe complication, and due to their infrequent occurrence, each case is interesting to report in order to improve the diagnosis and management of this uncommon tumor. Papillary fibroelastoma most commonly involve the aortic valve, followed by the mitral valve. Less frequent sites of involvement include mitral chordae tendinae, right atrial endocardium, endocardial surface of both ventricles including papillary muscles and interventricular septum (5, 16). We are presenting a very rare case of papillary fibroelastoma on mitral valve tendinae. Bajaj et al. reported papillary fibroelastomas attached to the interventricular septum (17).

Hakim et al. presented papillary fibroelastoma of the pulmonary valve (18). Zhang et al. reported four cases of papillary fibroelastoma with different locations of the left atrium,mitral valve and aortic valve (19). Generali et al. reported pulmonary valve papillary fibroelastoma (20). Surgical intervention is the definitive treatment for cardiac fibroelastoma and should be done in all symptomatic or asymptomatic patients but have mobile lesions for prevention of complications (10). 

In this paper, we report the uncommon location of a PFE without valvular involvement. Resection of these tumors might lead to mitral regurgitation, and heart failure is unavoidable in some valvular and chordae tendinaes involvement as in some previous cases, therefore, valvular repair or replacement may be required. In our case, although the tumor was attached to three chordae tendinaes and was not pedanculated, the tumor was completely resected from mitral valve chordae with sharp dissection. Fortunately, chordae tendinae were preserved successfully without destruction. After 7 months follow-up, our patient had no symptoms and at last TTE showed normal chambers with normal mitral valve without regurgitation or recurrence of tumor. In conclusion, our patient was presented with atypical chest pain and palpitation. We suggest more attention and echocardiographic assessment for evaluating these common symptoms especially in young patients, although these signs and symptoms occurred more often after common ischemic heart diseases. 
